# Cell-Free DNA in the Pathogenesis and Therapy of Non-Infectious Inflammations and Tumors

**DOI:** 10.3390/biomedicines10112853

**Published:** 2022-11-08

**Authors:** Györgyi Műzes, Bettina Bohusné Barta, Orsolya Szabó, Vanessza Horgas, Ferenc Sipos

**Affiliations:** Department of Internal Medicine and Hematology, Semmelweis University, Szentkirályi Street 46, 1088 Budapest, Hungary

**Keywords:** cell-free DNA, CpG oligonucleotides, inflammation, autoimmunity, tumor, absent in melanoma-2, Toll-like receptor 9, cyclic GMP–AMP synthase, stimulator of interferon genes

## Abstract

The basic function of the immune system is the protection of the host against infections, along with the preservation of the individual antigenic identity. The process of self-tolerance covers the discrimination between self and foreign antigens, including proteins, nucleic acids, and larger molecules. Consequently, a broken immunological self-tolerance results in the development of autoimmune or autoinflammatory disorders. Immunocompetent cells express pattern-recognition receptors on their cell membrane and cytoplasm. The majority of endogenous DNA is located intracellularly within nuclei and mitochondria. However, extracellular, cell-free DNA (cfDNA) can also be detected in a variety of diseases, such as autoimmune disorders and malignancies, which has sparked interest in using cfDNA as a possible biomarker. In recent years, the widespread use of liquid biopsies and the increasing demand for screening, as well as monitoring disease activity and therapy response, have enabled the revival of cfDNA research. The majority of studies have mainly focused on the function of cfDNA as a biomarker. However, research regarding the immunological consequences of cfDNA, such as its potential immunomodulatory or therapeutic benefits, is still in its infancy. This article discusses the involvement of various DNA-sensing receptors (e.g., absent in melanoma-2; Toll-like receptor 9; cyclic GMP–AMP synthase/activator of interferon genes) in identifying host cfDNA as a potent danger-associated molecular pattern. Furthermore, we aim to summarize the results of the experimental studies that we recently performed and highlight the immunomodulatory capacity of cfDNA, and thus, the potential for possible therapeutic consideration.

## 1. Introduction

The discovery of cell-free deoxynucleic acids (cfDNA) in the sera of cancer patients in 1948, is attributed to Mandel and Métais [1]. Later, a correlation was observed between the concentration of cfDNA and the development of systemic lupus erythematosus [2]. The use of cfDNA in the diagnosis of tumors began in 1977, but was not very effective, due to the limitations of the existing technology [3]. The real-time polymerase chain reaction allowed the detection of RhD and the fetal sex in maternal plasma in 1997 [4]. The real expansion of non-invasive fetal genetic disease detection began in 2011, with the introduction of massive parallel sequencing [5]. Approximately fifty percent of prenatal genetic examinations are performed today via so-called non-invasive prenatal testing (NIPT) [6]. Recently, the spread of liquid biopsies and the increased demand for screening, as well as monitoring disease activity and the therapeutic response, made it possible for cfDNA research to be revived. Though the analysis of the 5′ ends of extracellular DNA demonstrated the unique character of extracellular DNA (i.e., definitely not being a junk molecule) [7], investigations primarily focus on the role of cfDNA as a biomarker, and research regarding the immunological properties of cfDNA, such as its potential immunomodulatory or therapeutic benefits, is still in its infancy. In this review, we aim to summarize the findings of recent experimental studies and highlight the immunobiological effects of cfDNA, as well as the potential for future therapeutic considerations in the context of sterile inflammation and the onset of cancer. 

## 2. Origin, Release, Amount, and Clearance of Cell-Free DNA

Though cfDNA is ubiquitously present in human body fluids [8], and many aspects of its molecular source are known, research to uncover the unknown factors in its origin is growing and may never end. Except for the exogenous sources of cfDNA [9], many possible endogenous origins and related mechanisms have been proposed [10]. Regarding the cellular source of cfDNA, tumorous (i.e., local and circulating tumor cells, micrometastases, and cells of the tumor microenvironment) and non-tumorous cells (e.g., muscle cells, epithelial cells, ovum cells, bone cells, myeloid and lymphoid cells) can be distinguished [10]. 

The mechanisms responsible for cfDNA release are quite diverse. On the one hand, cell death and clearance mechanisms (i.e., apoptosis, necrosis, pyroptosis, mitotic catastrophe, autophagy, phagocytosis, oncosis, NETosis, and DNA excision repair damage) are partly responsible for the release of cfDNA [11,12]. On the other hand, the active release is also possible via macromolecular structures (DNA-protein complexes, extracellular traps), micronucleation induced by genome instability (extrachromosomal circular DNA), or microvesicles (exosomes) [13,14,15].

Different data is available on the amount of human cfDNA in circulation since no standardized methods exist. The choice of matrix (i.e., serum, plasma, urine, cerebral fluid, etc.), the mode of sample collection (e.g., EDTA-containing tubes or CellSave tubes, etc.), the parameters of centrifugation (i.e., speed, temperature, duration), types of isolation kits, and cfDNA storage conditions can all influence the measurement results [16]. In general, the level of cfDNA in the healthy population is lower, as compared with diseased people. According to the latest data [17], the normal human plasma cfDNA concentration can be as high as 500 ng/μL. In cases of advanced cancers [18,19,20], autoimmune [21,22,23,24,25], inflammatory [26], traumatic [27,28], post-transplantation [29] or infectious diseases [30,31] usually a more increased amount is detected. In addition, cfDNA levels could also be increased, due to vigorous physical exertion (such as intense sports, e.g., half marathon, ultramarathon, TRX exercises) [32,33] and pregnancy [34]. Fetal cfDNA, which is primarily produced by placental trophoblast cells during pregnancy [35], is detected in the maternal circulation, as early as in the first trimester, accounting for 10 to 15% of the total cfDNA concentration [36]. 

The concentration of cfDNA can increase, not only under the previously mentioned conditions, but also as a result of an increase in release. Ineffective clearance mechanisms could also contribute significantly to the elevated levels of circulating cfDNA. Extracellular nuclease homologs, DNase I and DNase I-like III (DNase I L3), are responsible for the efficient degradation of both free and protein-bound DNA [37]. The enzyme’s ability to recognize and degrade DNA could be influenced by the abnormalities of DNase I activity (e.g., low serum DNase I activity [38], elevated serum levels of DNase I inhibitors [39], novel mutations in the enzyme [40]), molecules that interact with DNA [41], anti-DNase antibodies [42,43], and deficiencies in DNase I activating cofactors, such as the complement component C1q [44], TREX1 DNase [45], serum amyloid P component [46], IgM [47], C-reactive protein [48], and mannan-binding lectin [49]).

## 3. Cell-Free DNA as a Molecular Marker or a Diagnostic Tool

**Figure 1:**
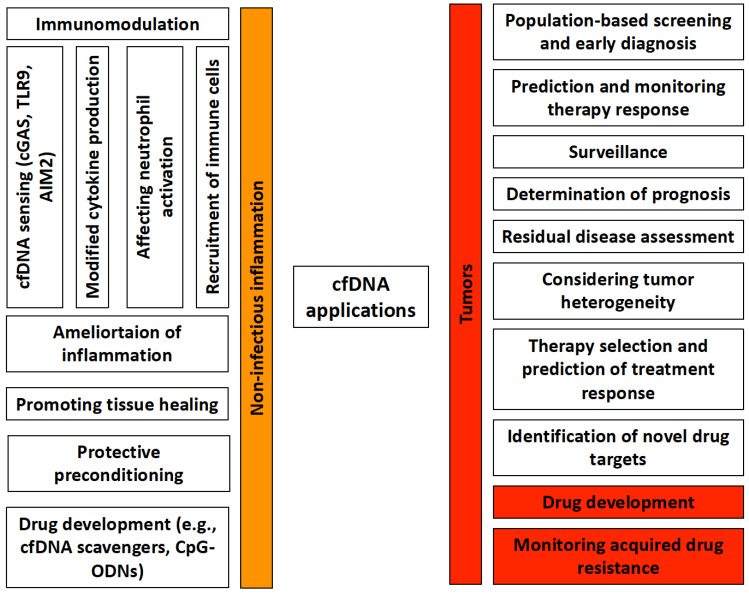
Potential clinical and experimental applications of cfDNA in non-infectious inflammations and tumors. cfDNA has an immunomodulatory effect in non-infectious inflammations, which is mediated by cfDNA sensing, changes in cytokine production, neutrophil activation, and effects on other immune cells. cfDNA by itself can reduce inflammation, promote tissue healing, and is also suitable for protective pretreatments. In addition, it can serve as a promising starting point for drug development. In tumors, it can play a significant role in population-level screenings, early diagnosis, and therapeutic response determination. In addition to being able to predict the course of the disease, it can also be used to determine any residual disease after treatment. It provides information on the heterogeneity of tumor cells, thereby facilitating the selection of the most effective treatment. It can serve as a basis for drug development. Furthermore, it allows for the monitoring of acquired drug resistance.

Based on its close association with a number of human physiological and pathological conditions, the clinical utility of cfDNA as a noninvasive, reliable, sensitive, and rapid diagnostic marker is continuously the subject of intense research (Figure 1).

### 3.1. cfDNA in Prenatal Diagnosis

Prenatal genetic testing is among the fields in which the utilization of circulating cfDNA has had the most success and is still widely used [50]. NIPT became a clinical reality in 2011 [51]. The fetal-derived cfDNA can be detected as early as the 4th week of gestation [52], and it is quickly eliminated from the maternal bloodstream after delivery [53], emphasizing its pregnancy specificity. The first clinical applications were limited to the identification of alleles present in the fetus and not in the maternal genome (i.e., paternal or de novo mutations) [54]. In contrast, the establishment of autosomal recessive or maternally transmitted autosomal dominant disorders, has been much more complicated, even though several studies have succeeded in determining the exclusion of paternal alleles in recessive conditions. However, thanks to the continuous development of technology, it is now possible to determine the sex, RhD, and blood group of the fetus [55]. In addition, cfDNA also allows the identification of fetal aneuploidies and specific microdeletions [56]. Though the measurement of fetal cfDNA is noninvasive, widely applicable, and available early in pregnancy, it has some limitations. The current detection of aneuploidy is limited to common trisomies [57], therefore the karyotype determination is still necessary. Furthermore, fetal cfDNA determination is currently irrelevant for diagnosing monogenic disorders, autosomal recessive, or X-linked diseases [58]. So, the technique needs to be improved. 

### 3.2. cfDNA in Tumors

The growing interest in tumor-related cfDNA is a direct result of its potential use as a liquid biopsy tool, which has great promise for a wide range of clinical applications [59,60]. Even if a surgical biopsy/histology remains the gold standard for cancer diagnosis and treatment, it has some disadvantages (i.e., it is invasive and provides temporary static images of malignancy) [61]. In contrast, tumor cfDNA detection enables the real-time longitudinal monitoring of cancer, along with capturing tumor heterogeneity [62,63,64]. Moreover, in the last few years, there has been a strong concordance between plasma and tissue-based genomic studies, encouraging the exploration of their potential clinical utility [65,66,67,68]. Tumor cfDNA has received a lot of attention in early tumor detection for several types of cancer [69], however, the process for purification and handling of cfDNA is not yet standardized, and numerous preanalytical variables, such as the purification kits, blood collection tubes, and centrifugation regime, may affect cfDNA’s yield and analysis [70,71]. Thus, more sensitive and reproducible techniques are required. For screening, the combined use of tumor cfDNA and conventional tumor markers seems to be an optimal application [72,73,74]. Several studies have demonstrated that in several types of cancer tumors, cfDNA is suitable for detecting minimal residual disease postoperatively or after chemotherapy [75,76,77], which suggests that it has a high prognostic value with the ability to predict the disease recurrence. The genotyping of tumor cfDNA is useful, not only in choosing the optimal treatment and dynamically monitoring the therapeutic responses [78], but it can also reveal the genetic causes of malignancy progression and therapy resistance, as well [78]. Applications of tumor cfDNA in this direction seem feasible and are close to being introduced into clinical practice.

### 3.3. cfDNA in Non-Tumor Disorders

In pathological conditions, such as autoimmune diseases [79,80,81,82], stroke [83], myocardial infarction [84,85], and allograft transplant rejection [86], there is substantial interest in the investigation of cfDNA’s clinical utility, but no real medical applications have been developed yet. Elevated levels of cfDNA in SLE patients appear to be associated with antibody titers and active lupus nephritis [82,87], but its correlation with disease activity, as well as the diagnostic and prognostic values, remains uncertain [83,87]. In rheumatoid arthritis (RA) patients, the serum level of cfDNA seems to be quite varied [81,88,89]. The cfDNA concentration of synovial fluid is several times higher than that in circulation, indicating the importance of local inflammation in the cfDNA release [90]. In RA patients, the dynamics of cfDNA appear to be independent of the conventional diagnostic markers, ACPA and RF. Though studies suggest the biomarker potential of cfDNA, further studies with large patient cohorts are necessary to analyze the dynamics of cfDNA in RA, in relation to the disease progression and drug effects. 

In cases of stroke, the dynamically determined blood levels of cfDNA appear to be a valid and reliable option for establishing prognostic and diagnostic criteria [91]. While cfDNA has performed well in a number of studies as a stroke biomarker [92,93], none of the so-called stroke biomarkers identified to date have proven useful in medical practice, and there is still a long way to go before its clinical application, either as a standalone marker or as part of a biomarker panel. 

cfDNA testing provides an alternative method for monitoring myocardial ischemia and has potential clinical applications for identifying high-risk individuals [94]. However, several biological and technical obstacles were recognized in cell-free DNA testing [95], including the lack of specificity and unsuitable kinetics for early cardiomyocyte damage, the long turnaround time and limited bandwidth, the need for specialized equipment and specialized staff, the absence of standardized or harmonized analytical techniques, the indirect expenses, and the high susceptibility to preanalytical variables [95]. Therefore, it seems acceptable to conclude that the analysis of cell-free DNA in diagnosing myocardial ischemia is not yet ready for commercialization.

In organ transplantation, the diagnostic role of cfDNA has been extensively studied in heart, kidney, and lung transplantations [96]. However, only one study exists on this topic in liver transplantation [97]. Despite the many results supporting the association between the amount and kinetics of donor-derived cfDNA and transplant organ rejection, neither the US Food and Drug Administration nor the European Medicines Agency has approved the use of cfDNA in this context. Based on the objections, clarification is needed on both the threshold and kinetics.

## 4. Recognition and Immunomodulatory Role of Cell-Free DNA

In addition to being a biomarker and a diagnostic tool, cfDNA has been shown experimentally to have an immunomodulatory effect. It can influence the initiation, progression, or amelioration of inflammation. The presence of self-DNA in the nucleus and mitochondria is necessary for the maintenance of self-tolerance. However, following nuclear or mitochondrial damage, self-DNA enters the cytosol under stress conditions. In the apparent lack of infection, the inflammatory response is likely triggered by the production of endogenous alarmins, known as danger-associated molecular patterns (DAMPs), which trigger immune responses via pattern-recognition receptors (PRR). Cell-free DNA could act as a DAMP [98,99]. 

The recognition of cfDNA could be performed by the DNA-sensing receptor cyclic guanosine monophosphate (GMP)-adenosine monophosphate (AMP) synthase (cGAS), Toll-like receptor 9 (TLR9), or absent in melanoma-2 (AIM2)-like receptors (ALRs) [100].

The cGAS identifies cytosolic DNA and induces the interferon regulatory factor (IRF) 3-dependent interferon-beta (IFNβ) or type 1 interferons [101]. cGAS recognizes extracellular nucleosomes as well, because they have a higher binding capacity than double-stranded DNA (dsDNA) [101]. Stimulator of interferon genes (STING) participates in the cGAS signaling pathway in response to the recognition of cytosolic DNA [102,103]. The cGAS optimally recognizes 36 base pair long dsDNA (or longer) to activate the cGAS-STING-mediated effectors to generate type 1 interferons and other nuclear factor (NF)-kB-dependent cytokines, regardless of the sequence [100,104,105]. In addition to NF-kB, mitogen-activated protein kinase (MAPK), and signal transducer and activator of transcription 6 (STAT6) activation, STING stimulates the autophagosome formation by facilitating the microtubule-associated protein 1A/1B-light chain 3 (LC3) puncta formation and the autophagy related (Atg) 9a, upon recognizing the cytosolic dsDNA [106,107,108,109]. Beclin-1 (BECN1) interacts with cGAS to restrict cGAMP formation in response to cytosolic dsDNA, by inhibiting the interaction between cGAS and dsDNA. The interplay between cGAS and BECN1 results in the release of Rubicon (negative regulator of autophagy) from BECN1, which activates the class III phosphatidylinositol 3-kinase function to induce autophagy and hence eliminate cytosolic dsDNA [110]. One of the main functions of autophagy is to eliminate cfDNA without causing inflammatory damage. The defected autophagy enhances the inflammatory recognition of cfDNA by various cytosolic PRRs [110].

TLR9 is present in the endoplasmic reticulum (ER), during normal physiologic stages. However, when cytosolic cytosine-phosphate-guanine (CpG)-DNAs or self-DNA enter the endosome or endolysosome, TLR9 migrates to these organelles and recognizes them as essential DAMPs [111,112]. In order to cause inflammation and inflammatory diseases, the TLR9 activation induces a myeloid differentiation primary response 88 (MyD88)-dependent downstream signaling pathway that activates the IRF3-based type1-interferon production and NF-kB-mediated pro-inflammatory cytokine production [113]. The Toll-interleukin-1 receptor (TIR) domain of MyD88 activates the interleukin 1 receptor-associated kinase (IRAK)-4 and IRAK-1 [114,115]. IRAK-4 recruits the tumor necrosis factor receptor-associated factor 6 (TRAF6) to activate the transforming growth factor-β-activated kinase 1 (TAK1) [116]. TAK1 phosphorylates the IκB kinase (IKK) complex via the K63-linked ubiquitination of the NF-kB essential modulator (NEMO), which is crucial for the NF-kB, IRF3, and MAPK signaling [117]. TLR9 recognizes two types of DNA (i.e., pathogen-derived and self-DNA). It was shown that the nucleotide sequence, length, and dimerization properties of synthetic CpG-oligodeoxyribonucleotides (ODNs) and cfDNAs determine their tendency to bind and activate TLR9 [118,119,120]. The intracellular compartmentalization of TLR9 is a mechanism for discriminating between self- and non-self-DNAs [121]. Their binding results in an increase in the dimerization and activation [121]. 

Platelets are known to express PRRs, which can be triggered upon interaction with DAMPs [122]. Platelets from both murine and human hosts express TLR9 [123,124], which is of importance because, in addition to their hemostatic function, platelets play a crucial role in bridging innate and adaptive immunological responses [122]. Platelet activation results in the platelet production of P-selectin, which enables platelets to attach to other cells, such as granulocytes, leading to the granulocyte activation and recruitment to sites of tissue damage. Platelets are activated by cfDNA, which contributes to the creation of neutrophil extracellular traps (NETs) [122].

**Figure 2:**
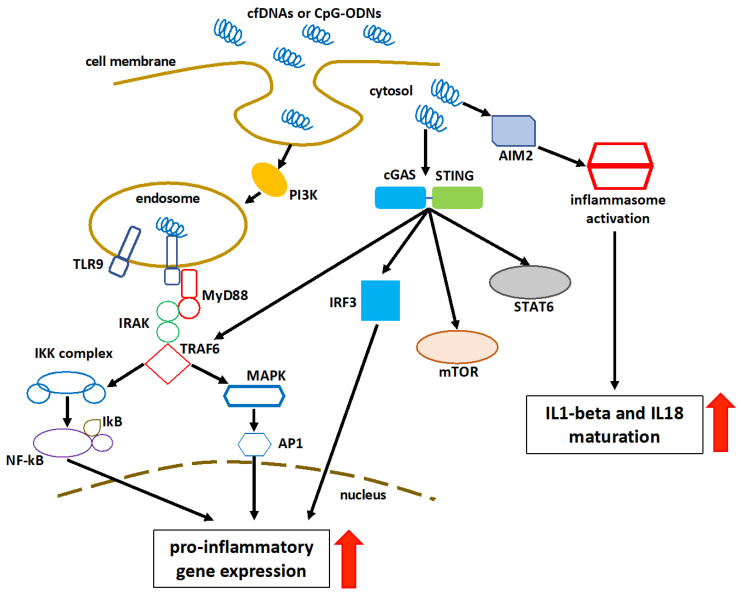
Schematic representation of cell-free DNA sensing and consequent pathway activation. Class III PI3K promotes the internalization of cfDNA and CpG-ODNs into TLR9-containing endosomal vesicles. The intracytoplasmic activation signal is transmitted via the interaction of cfDNA and TLR9. MyD88 is recruited to the Toll–interleukin-1 receptor domain of TLR9, followed by the activation of the IRAK–TRAF6 complex. This activates both the MAPK and the inhibitor of IKK complexes, resulting in the overexpression of transcription factors, such as NF-kB and AP1. cGAS-mediated detection of cytosolic DNA initiates a STING-dependent reaction. The cGAS-STING pathway can also activate IRFs, mTOR, STAT6, and MAPK in a direct or indirect way. In the cytosol, AIM2 binds to the double-stranded DNA, resulting in the creation of the AIM2 inflammasome. This results in the activation of caspase 1, the maturation of proinflammatory cytokines IL-1β and IL-18, and finally pyroptosis. AP1: activator protein 1; red arrows: upregulation.

AIM2 is an ALR that is activated upon recognizing and binding to self-DNA entering the cytosol, as a result of cellular damage and exosomes containing self-DNA [125]. AIM2 efficiently activates in response to 80–300 base pair self-DNA [126,127]. The HIN (hematopoietic expression, interferon-inducible nature, and nuclear localization) domain of AIM2 recognizes cytosolic DNA, and its pyrin domain (PYD) interacts with the PYD of ASC (apoptosis-associated speck-like protein containing a C-terminal caspase recruitment domain) to form an inflammasome complex that converts procaspase 1 (pro-CASP1) to CASP1 [126,128]. CASP1 releases IL-1 and IL-18 from their preforms [128]. CASP1 also cleaves the Gasdermin D (GSDMD) linker region, finally mediating the release of IL-1 and IL-18 from cells. Additionally, the K^+^ efflux from the GSDMD pore inhibits the cGAS activity and the cGAS-STING-mediated release of type 1 IFN, as well as induces pyroptosis [129,130,131]. The AIM2-induced GSDMD functions as a negative regulator of type 1 interferon production mediated by cGAS-STING [125]. In addition, the AIM2-ASC inflammasome inhibits the STING-TBK1 (TRAF family member-associated NF-κB activator-binding kinase 1) interaction required for the IRF3-dependent release of type 1 interferons [131,132]. In the absence of certain cytosolic DNAs, AIM2 remains inactive [132]. A schematic representation of the cfDNA recognition and consequent pathway activation is shown in Figure 2.

## 5. Cell-Free DNA-Mediated Inflammatory Disorders

The CfDNA-induced inflammation is a pathogenic factor in several diseases. The activation of TLR9, which releases type 1 interferons via cfDNA, increases liver inflammation in metabolic liver diseases (e.g., non-alcoholic steatohepatitis /NASH/ or non-alcoholic fatty liver disease /NAFLD/) by accelerating the non-apoptotic death of hepatocytes [133]. The modulation of high mobility group box 1 (HMGB1) in NASH prevents weight gain and liver inflammation, indicating that TLR9 recognizes the self-DNA bound to HMGB1 in C57BL/6 mice fed a high-fat diet [134]. Dietary steatohepatitis is exacerbated by the TLR9 activation, which upregulates the AIM2 expression and the IL-1β production [135]. In ischemia-reperfusion-induced hepatitis, the AIM2 stimulation in Kupffer cells in response to oxidized mitochondrial cfDNA, also plays a critical role [131,136]. In NASH and NAFLD [137,138], as well as in alcoholic liver disease [139,140], the cGAS-STING system (mainly in Kupffer cells) recognizes mitochondrial cfDNA as a DAMP, which can lead to inflammation and fibrosis.

The TLR9-mediated identification of cfDNA plays a crucial role in the inflammation and insulin resistance index associated with obesity. The level of circulating endogenous cfDNA rises in obese individuals, patients with visceral obesity, and mice fed a high-fat diet [141,142]. The increased circulating endogenous host-derived cfDNAs enhance the accumulation of pro-inflammatory M1 macrophages in adipose tissues upon recognition by TLR9 [142].

Mitochondrial cfDNA may also play a role in obesity, caused by a high-fat diet, since the knockout of STING prevents obesity in mice [143]. In adipocytes, the stress-induced mitochondrial cfDNA release activates phosphodiesterases (PDE3B/4), which causes a decrease in the cAMP levels and the inhibition of protein kinase A signaling, ultimately resulting in a decreased thermogenesis [144]. When a high-fat diet containing palmitic acid is used, the mitochondrial cfDNA-induced cGAS-STING signaling also occurs in endothelial cells, which leads to adipose tissue inflammation, obesity, glucose intolerance, and insulin resistance [144,145]. 

Atherosclerosis is also related to the cfDNA-mediated TLR9-signaling. According to studies conducted on animals, the angiotensin II infusion increases the plasma concentration of self-DNA recognized by TLR9 expressed on immune cells, such as macrophages, which secrete proinflammatory cytokines promoting atherogenesis in the aortic arch [146]. By turning on the p38MAPK pathway, the TLR9 activation in apolipoprotein E-deficient macrophages worsens the inflammation [146]. Electronic cigarette use has also been demonstrated to raise the level of mitochondrial cfDNA in the blood and induce the expression of TLR9, both of which increase the expression of proinflammatory cytokines in monocytes and macrophages and thereby contribute to the development of atherosclerosis [147]. Circulating cfDNA binding to TLR9 increases, in conjunction with HMGB1 binding [148]. Studies have shown that patients with coronary artery disease (CAD) have higher circulating levels of HMGB1, which is associated with the non-calcified plaque burden in stable CAD patients [148,149]. HMGB1 levels are also linked to CAD in non-diabetic and type 2 diabetes mellitus patients [149,150]. The AIM2 activation may also be involved in the pathophysiology of atherosclerosis. The intravenous administration of poly(dA:dT) (deoxyadenylic-deoxythymidylic) acid, a synthetic analog of the canonical right-handed DNA helix, also known as B-DNA, results in the release of AIM2-dependent proinflammatory cytokines that disrupt the carotid artery reendothelialization [151]. Furthermore, subcutaneous poly(dA: dT) injection activates AIM2, causing the atherosclerotic plaque formation, an increased reactive oxygen species (ROS) production, and the endothelial microparticle release in ApoE-/-mice [151]. 

Murine models have demonstrated that endosomal TLRs (i.e., TLR7 and TLR9) play a crucial role in SLE and related systemic autoimmune diseases [152,153]. The spontaneous generation of autoantibodies against self-DNA in autoreactive B cells is facilitated by the TLR9 signaling coupled with the B cell receptor signaling [154,155]. The TLR9 deficiency, moreover, worsens SLE, due to the profound activation of lymphocytes and plasmacytoid dendritic cells, as well as an increase in serum immunoglobulin G and IFNα levels [152]. In addition, TLR9 has no effect on the progression of lupus nephritis in susceptible mice [152].

The role of STING in SLE is, however, controversial [156]. Loss of function mutations in the extracellular DNAse1L3 lead to the accumulation of DNA/RNA-associated microparticles in the circulation [157,158]. The genetic deletion of DNAse1L3 in mice causes a disease that is similar to SLE [159]. This disease is caused by a mechanism that depends on TLR7 and TLR9 but not STING [160]. Experimental findings indicate that SLE is driven by extracellular DNA delivered to endosomal TLRs via receptors, such as the B cell receptor, LL37, or FcγRs [156], whereas monogenic autoinflammatory diseases (e.g., Aicardi–Goutières syndrome; type I interferonopathy due to a DNase II deficiency) are driven by the abnormal accumulation of DNA in the cytosol, which is detected by the cGAS/STING pathway [161,162].

The cGAS-STING signaling induced by cfDNA also plays a pivotal role in a variety of sterile inflammatory diseases in humans (including ataxia-telangiectasia, familial amyotrophic lateral sclerosis, frontotemporal dementia, STING-associated vasculopathy with the onset of infancy, erosive inflammatory arthritis, psoriasis, Bloom syndrome, and Huntington’s disease) [138,163,164,165] and in murine experimental autoimmune encephalitis [164]. 

Alterations of the negative regulators (e.g., protein phosphatase 6 catalytic subunit of protein phosphatase 6, immunity-related GTPase M, Myb-like, SWIRM, and MPN domains 1 protein /MYSM1/) of the cGAS-STING signaling pathway could also lead to the development of human autoimmune diseases [166,167,168,169]. Monocytes isolated from the peripheral blood of SLE patients express less MYSM1 but produce more type 1 interferons [169]. MYSM1 binds to STING and inhibits the cGAS-STING signaling pathway [169]. Furthermore, MYSM1 inhibits inflammation mediated by NOD2 (nucleotide-binding oligomerization domain-containing protein 2), CARD15 (caspase recruitment domain-containing protein 15), or IBD1 (inflammatory bowel disease protein 1), by inactivating the receptor interacting protein 2 (RIP2) complex, thereby preventing the formation of the NOD2-RIP2 complex, which is essential for the inflammatory signaling pathway [170].

## 6. Cell-Free DNA as a Possible Modulator of Sterile Inflammation

In light of what has been discussed thus far, it is clear that the recognition of cfDNA by PRRs plays an important part in the pathogenesis of a wide variety of sterile inflammatory diseases. Based on these, it makes sense to try using cfDNA as an immunomodulator to change the course of inflammation (Figure 1). 

Inflammatory bowel diseases (IBDs) are caused by a dysfunctional mucosal immune response to intestinal microbiota and other luminal antigens. Traditional IBD treatments primarily target aberrant immune responses and inflammatory cascades. However, some of these treatments have limited efficacy and can cause severe side effects. Dextran sulfate sodium (DSS)-colitis, an experimental mouse model of inflammatory bowel disease (IBD), is particularly useful for studying the contributions of the innate immune system (including TLR9-signaling) to the pathomechanism and therapy of colitis [171,172]. The amount of cfDNA is correlated with the severity of the intestinal inflammation in mice with chemically-induced colitis [173]. In experimental murine colitis, the beneficial therapeutic effects of orally administered immunostimulatory DNA sequences and their synthetic oligonucleotide analogs have already been demonstrated [174,175]. In addition, the intraperitoneal (ip) administration of immunostimulatory TLR9-agonist DNA sequences protects mice from DSS-induced colitis via the induction of indoleamine 2,3 dioxygenase-1 [176,177]. It is commonly reported that an ip injection is as effective as an intravenous (iv) injection. However, it has also been demonstrated that the pharmacokinetics of ip DNA analogues are different from those of iv DNA analogues [178,179]. The local administration of a TLR-agonist synthetic oligonucleotide sequence (DIMS0150) in humans has demonstrated clinical efficacy by restoring the glucocorticoid sensitivity, but the colonoscopy-based therapy administration is challenging [180,181,182,183,184]. Consequently, an easier and more convenient route of drug administration (orally or parenterally) will be necessary in the future. To our knowledge, we were among the first to investigate the biological effects of iv administered cfDNA in a therapeutical setting in a mouse model of DSS-colitis.

In a murine DSS-colitis experiment, in which the therapeutic efficacy of iv-administered cfDNA was evaluated [185], we discovered that, under inflammatory conditions, the systemic administration of colitis-derived cfDNA can decrease the clinical and histological severity of DSS-induced murine colitis, possibly by modifying the proinflammatory cytokine expression and the TLR9-related signaling. The subsequent presence of a markedly inflammatory environment, likely caused by the induction of severe colitis, may result in cfDNA with the potential to promote the suppression of inflammation and enhance tissue regeneration.

The connection between TLRs and autophagy, in response to DAMPs has been verified by a number of studies [186,187]. This regulatory cross-talk between them partly serves to trigger the innate immune system. Concerning the TLR9-autophagy linkage in murine DSS-colitis, we have demonstrated for the first time that the final, sometimes beneficial effect of iv administered cfDNA on the autophagy response, depends on two factors: i., the origin of the cfDNA (i.e., inflammatory or non-inflammatory) and ii., the local immunobiological milieu (i.e., inflammatory or not), as well [188].

Based on how cfDNA affects the immune system, it is evident that many studies have been performed to find out what role the DNA-sensing pathway plays in inflammation, by generating and using synthetic ODN sequences. There are numerous subtypes of TLR9 activating/inhibitory synthetic ODNs [189,190]. Type-A CpG-ODNs frequently form large multimeric aggregates spontaneously and are consequently retained in the early endosomes of plasmacytoid dendritic cells (pDCs) for relatively long periods, resulting in the prolonged activation of the signal-transducing complex and the robust IFNα production. Type-B CpG-ODNs, in contrast, stay monomeric and are rapidly transported from early to late endosomes, making them potent B and NK cell stimulators. Type-C ODNs exhibit mixed characteristics; they function as potent stimulators of pDCs’ IFNα production, the antigen presenting cell activation and maturation, indirect NK cell activation, and direct B cell stimulation [189].

In the early stages of RA, it appears that the T cell-dependent B cell activation is necessary for the rheumatoid factor (RF) production. Nonetheless, later in the course of the disease (i.e., after five years), when the majority of patients received immunosuppressive disease-modifying drugs, a dissociation between the T cell reactivity and the RF status was observed [191]. This suggests that T cells are no longer needed for B cells to generate RF. RF and cytokines produced by B cells and monocytes are associated with an increase in the later phase of RA, as a result of the CpG-ODN treatment of peripheral mononuclear blood cells of RA patients. This suggests that B cells (including the RF-producing B cells) may lose their dependence on the T cells. The TLR agonistic CpG-ODNs can maintain the polyclonal memory B cell populations [192], and the patient’s own IgG/cfDNA complexes can activate the B cells effectively [155]. Consequently, as RA progresses, the cfDNA-dependent B cell antibody production increases. It has also been demonstrated that the injection of the human TLR9 agonist CpG-ODN2006 into the articular cavity of mice, led to the development of acute arthritis [193]. Similar to CpG-ODN2006, a CpG motif-rich RA-associated ODN induces joint arthritis [90,194].

In a murine model of Sjögren’s syndrome [195], it was demonstrated that activation of TLR9 by BL-7040 (an antisense ODN against acetylcholinesterase mRNA) results in the non-canonical activation of NF-kB, thereby enhancing the salivary function and suppressing inflammation. 

Furthermore, by stimulating the Th1 immune response, early (i.e., one week of age) the CpG-ODN treatment prevented the development of the Th2-driven scleroderma-like syndrome in tight-skin mice [196]. However, delaying the CpG-ODN treatment until six weeks of age was ineffective in preventing the skin disease [196].

Using CpG-ODNs, the pathogenetic role of cfDNA sensing was also highlighted in antineutrophil cytoplasmic antibody (ANCA)-associated vasculitides (AAVs). Circulating ANCA-autoreactive B cells are present in patients with AAVs. Upon stimulation with unmethylated CpG-ODNs, these cells produced ANCA [197]. It cannot be ruled out that stimulating ANCA-autoreactive B cells with CpG-ODNs of other cfDNAs may be a link between infection and AAVs.

The administration of an immunoregulatory GpG-ODN (with a single base switch from CpG to GpG) can reduce the severity of Th1/Th17-mediated EAE in mice [198]. The GpG-ODN inhibited Th1 cytokine production and decreased the expression of the co-stimulatory and MHC molecules by antigen-presenting cells. The GpG-ODN changed the phenotype of autoreactive Th1 cells to a protective Th2 phenotype and the isotype switching of the autoreactive B cells to a protective IgG1 isotype [199]. In addition, the protective mechanism of the GpG-ODN treatment in the NZB/W lupus nephritis model was demonstrated. This mechanism modifies the cytokine profiles of the T cells and activates the B lymphocytes via the inhibition of TLRs, such as TLR-7 and TLR-9 [200].

Based on the above, it can be seen that the immunomodulatory, anti- or proinflammatory effect of cfDNA and synthetic ODNs depends not only on the structure and origin, but also on the time of application (i.e., early vs. later age of a given disease). Furthermore, there is evidence that applying cfDNA as a “preconditioning treatment” before the initiation of inflammation, can ameliorate the inflammatory process and reduce the degree of tissue damage. We found that preconditioning with a single iv dose of colitis-derived cfDNA ameliorated the clinical and histological severity of murine DSS-colitis, as compared to cfDNA of non-colitic origin [201]. In this experimental setting, the TLR9-signaling and inflammation-related gene expressions were altered in a clinically favorable manner. Additionally, in continuation of this experimental setup, we also found that the preconditioning by iv colitic cfDNA, the activation of cell protective autophagy can be achieved in mice with DSS-colitis [202].

## 7. Cell-Free DNA in Tumors

Since the landmark works of Colotta [203], Hanahan, and Weinberg [204,205], chronic inflammation has been recognized as a hallmark of cancer. Numerous studies have demonstrated that the concentration of cfDNA in the blood of patients with several types of tumorous diseases is elevated. One of the attributes of cfDNA is its ability to induce inflammation. Hence, it seems logical to investigate the carcinogenic role of cfDNA-induced inflammation.

Cell-free DNA sensing by TLR9 has dual-faced effects on tumor cells. In human colorectal cancer (CRC) tissues, the TLR9 overexpression was detected [206]. By adding colon cancer cell-derived cfDNA or the TLR9 agonist CpG-ODN2395 to CRC cell lines, researchers found that the TLR9-MyD88 signaling boosted cell growth, migration, invasion, and IL8 secretion [206]. It has been reported that cfDNA is released from breast cancer primarily through the active secretion and that cfDNA can stimulate the proliferation of hormone-receptor positive breast cancer cells by activating the TLR9-NF-kB-cyclin D1 pathway [207]. Contrarily, it has been demonstrated that host TLR9 after sensing tumor cfDNA modulates the anti-tumor immunity in response to chemotherapy. TLR9 promotes the maturation and migration of DCs from the tumor microenvironment to regional lymph nodes, where DCs activate tumor-specific cytotoxic T lymphocytes, leading to potent anti-tumor effects [208]. 

During normal mitosis, the nucleosome inhibits the cGAS activation, in response to dsDNA through a competitive inhibition, and cGAS-STING signaling is not fully functional [209]. A low level of cGAS-STING signaling causes phosphorylation and the accumulation of IRF3 during mitotic arrest. This does not increase the production of type 1 interferon, thus does not cause inflammation, but at the same time, it results in apoptotic cell death [209]. Some anti-cancer medications, such as taxol, paclitaxel, or taxane, function in this way [209,210]. Sometimes, the cGAS-STING overexpression in certain tumors decreases the inflammatory immune cell infiltration, resulting in a poor prognosis [210]. The cGAS downregulation in patients with lung adenocarcinoma is also associated with an increase in mortality [211]. Further evidence suggests that the cGAS-STING signaling is important in the immune environment of various tumor microenvironments [212,213]. The activation of the STING signaling pathway improves the immunotherapy’s protective effects [212] and increases the potent tumoricidal T cell-mediated immune response [213]. In mouse models, the nuclear paraspeckle assembly transcript 1 inhibits the cGAS-STING signaling and cytotoxic T cell infiltration into the tumor microenvironment, thereby promoting tumor growth [214]. 

AIM2 plays an antitumor role in tumor diseases independently of the inflammasome activation [100,215]. This can be confirmed in chemically induced colitis-associated cancer [215], hereditary nonpolyposis colorectal cancer [216], and cutaneous squamous cell carcinoma [217]. At the same time, AIM2 promotes non-small cell lung cancer tumor growth by modifying the mitochondrial dynamics [218,219]. AIM2 also has protumor effects in oral squamous cell carcinoma [220], the start and spread of benign prostate hyperplasia [221], and chemically-induced hepatic cell carcinoma (HCC) [222]. However, in HCC, AIM2 can also displays antitumor properties [223]. 

The genometastatic hypothesis [224] is accepted as a model that could explain the experimental data inconsistencies concerning the metastasis formation [225]. The ability of tumor-derived cfDNA, including fragments of oncogenes, to behave in similar fashion to oncoviruses, provides an alternative route for the metastasis spread [226,227,228]. This hypothesis has been strengthened by the discovery of a DNA-containing secretome and data proving the horizontal DNA transfer between numerous different in vitro cells and organisms [229].

To prove the existence of genometastasis, human CRC-derived cfDNA containing KRAS, TP53, and HBB gene mutation fragments was isolated [225]. Following 20 days of incubation with this cfDNA, the NIH-3T3 murine tumor cells devoid of this mutant gene pattern were injected subcutaneously into NOD-SCID mice. In aggressive tumors developed from the “transformed” murine tumor cells, the mutant KRAS gene sequences were identified. In a similar experiment with human adipose tissue stem cells as recipients of tumorous cfDNA, however, neither mutant forms of the studied genes nor the tumor formation were observed [225]. In addition, the role of tumor-derived cfDNA in the malignant transformation has been proven in other cell cultures and animal models as well [227,228,230,231]. 

Regarding the formation of NETs (in which granulocytes release their own DNA decorated with the pathogen catching and killing granules into the extracellular environment) [232], it has been shown that tumor development and metastasis are accompanied by the excessive NET formation, which enhances adhesion, invasion, and sometimes immune escape [233]. In addition to serving as a scaffold and a trapping element, DNA also acts via the CCDC25 receptor binding. Through the TLR4–TLR9 pathway, HMGB1 and neutrophil granule components, such as neutrophil elastase and ROS, activate tumor cells [234]. 

Alternatively, some evidence suggests that the NET deposition in tumor tissue may have a cytotoxic effect. Researchers discovered that NETs inhibited the growth of cancer cells by inducing apoptosis in Caco-2 and AML cells and by inhibiting the migration and survival of melanoma cells [235,236]. In a CT-26 murine intestinal adenocarcinoma model, the oncolytic vesicular stomatitis virus caused an inflammatory response that included blood clotting in the tumor’s blood vessels that was caused by neutrophils and probably spread by NETs [237]. Table 1 summarizes the putative impacts of cfDNA on tumor formation.

## 8. Modification of Cell-Free DNA to Influence the Tumor Cell Phenotype

Considering what has been discussed up to this point, it is clear that the structure and origin of cfDNA influence the biological effect it induces. By artificially modifying and then reintroducing self-DNA from tumor cells, the effects on tumor cell viability, metabolic activity, and proliferation were also investigated. In vitro cellular models lacked both the tumor microenvironment and the immune system of the tumor-bearing host. As a result, they enabled us to investigate the pathobiological effects of self-DNA administration in HT29 colon cancer cells.

Different degrees of self-DNA methylation and fragmentation, or their combination, influenced the gene expression of specific TLR9 signaling components and the expression of cytokeratin 20, which indicated the differentiation of undifferentiated HT29 cells [238].

In our next experiment [239], we provided evidence for a close existing interplay between the TLR9-signaling and the autophagy response with remarkable influences on the tumor cell survival in HT29 colon cancer cells, subjected to intact or modified self-DNA treatments. Interestingly, we also found the colonosphere formation with a strong cytoplasmatic CD133 immunoreactivity only in artificially hypermethylated DNA-treated HT29 cells. This phenomenon could indicate the survival of some cancer cells with a stem-like phenotype.

Further, we analyzed the complex interrelated roles of the hepatocyte-derived growth factor receptor (HGFR) inhibition and TLR9/autophagy signaling in HT29 cells subjected to modified self-DNA treatments [240]. We found that the metabolic activity and proliferation of the tumor cells altered according to the used DNAs and inhibitors. The non-modified genomic DNA, HGFR inhibitor, and chloroquine (autophagy inhibitor) reduced cell growth the most. In this situation, the proliferation-stimulating effect of the signal transducer and activator of transcription (STAT)3 overexpression might be offset by LC3B, demonstrating the HGFR-mTOR (mammalian target of rapamycin)-ULK1 (Unc-51 like autophagy activating kinase 1) involvement in the HGFR inhibitor-mediated autophagy. On the contrary, the hypermethylated DNA, TLR9 inhibitor, and HGFR inhibitor co-administration increased the tumor cell proliferation.

In another study [241], we discovered that the tumorous self-DNA and insulin-like growth factor 1 receptor (IGF1R) inhibition display anti-proliferative properties that can be suppressed by inhibiting the TLR9 signaling. The different effects of the IGF1R, TLR9, and autophagy inhibitors on the HT29 cell proliferation and autophagy suggest that the IGF1R-associated and non-IGF1R-associated autophagy machinery are “Janus-faced” regarding cell proliferation. Autophagy induced by self-DNA and inhibitors also resulted in the survival of CD133-positive HT29 stem-like cancer cells, which may play a role in the CRC recurrence.

## 9. Conclusions

PRRs that recognize cfDNA play a crucial role in maintaining cell homeostasis. Under normal conditions, the host’s DNA is found in the nucleus and mitochondria, which promotes the development of self-tolerance. When cells or tissues undergo stressful conditions, their genetic material is released into the cytosol, due to mitochondrial or nuclear damage. Thus, their recognition by PRRs becomes possible, and they represent a potential threat to the maintenance of homeostasis. The recognition of cytosolic DNA by TLR9 depends on its CpG content, while in the case of cGAS, it mainly depends on its length and curvature. AIM2 recognizes its own DNA as well as DNA from the pathogen, regardless of the CpG content. Since the activation of AIM2 inhibits the cGAS-STING signaling through the GSDMD production in a manner dependent on the type 1 interferon production, it is hypothesized that it has evolved as a negative regulator of excessive inflammation in response to the cGAS activation. Further studies are necessary to reveal the connections and unknown regulatory processes between the DNA-sensing receptors and sterile inflammation, as well as the development of cancer.

In addition to the biomarker and diagnostic roles of cfDNA, further investigation of the immunomodulatory and therapeutic effects of cfDNA is definitely necessary. The creation of new types of combined HGFR, IGF1R, autophagy, and/or TLR9 signaling inhibitors would play a significant role in the development of personalized antitumor therapies. Further research is required to investigate the biological effects of modified own DNA fragments, inhibitory or stimulating CpG-ODNs, as the methylation status or the length of the fragment can also influence the experimental results. However, the present experiments need to be further tested in other cell lines expressing TLR9 (and other DNA-sensing PRRs).

## Figures and Tables

**Table 1:** The possible effects of cfDNA on tumor formation.

Harmful and Beneficial Impacts of cfDNA in Tumors			
Protumor Effects		Anti-Tumor Effects	
**TLR9-MyD88 + ODN2395**	boosts cell growth, migration, invasion, and IL8 secretion [206]	**cfDNA sensing by TLR9**	modulates anti-tumor immunity in response to chemotherapy [208]
**TLR9-NF-kB-Cyclin D1**	stimulation of cell proliferation [207]		promotes maturation and migration of DCs to lymph nodes [208]
**cGAS-STING overexpression**	reduces intratumoral inflammatory cell infiltration [210]		activates tumor-specific cytotoxic T cells [208]
	leads to poor prognosis [210]	**low expression of cGAS-STING**	ameliorates inflammation [209]
**cGAS down-regulation**	increases mortality [211]		enhances apoptosis [209]
**cGAS-STING inhibition by NEAT1**	promotes tumor growth [214]	**STING activation**	improves the protective effects of immunotherapy [212]
**AIM2 cfDNA sensing**	modifies mitochondrial dynamics [218,219]		enhances T cell-mediated anti-tumor immunity [213]
**cfDNA containing secretome**	favors to supportive peritumoral milieu [229]	**AIM2 (regardless of inflammasome activation)**	favors tumor cell survival [100,215,216,217]
**horizontal DNA transfer**	favors to supportive peritumoral milieu [227,228,230,231]	**NET deposition**	displays cytotoxic effects [235,236]
**NET formation**	enhances adhesion, invasion, immune escape [232]		inhibits cell growth, migration, survival [235,236]
	serves as a scaffold and trapping element [234]		induces apoptosis [235]
**NET + TLR4-TLR9-HMBG1**	activates neutrophils [234]		
	activates tumor cells [234]		

## Data Availability

Not applicable.
